# A prospectus of plant growth promoting endophytic bacterium from orchid (*Vanda cristata*)

**DOI:** 10.1186/s12896-021-00676-9

**Published:** 2021-02-22

**Authors:** Sujit Shah, Krishna Chand, Bhagwan Rekadwad, Yogesh S. Shouche, Jyotsna Sharma, Bijaya Pant

**Affiliations:** 1 Central Department of Botany, Tribhuvan University, Kathmandu, Nepal; 2 Daffodil Agro Biological Research Center, Lalitpur, Nepal; 3 Current Address: Yenepoya Research Centre, Yenepoya (Deemed to be University), University Road, Deralakatte, Mangalore, 575018 Karnataka India; 4 National Centre for Microbial Resource, National Centre for Cell Science, Savitribai Phule Pune University Campus, Ganeshkhind, Pune 411021 India; 5 Department of Plant Science and Soil Science, Texas Tech University, Lubbock, Texas USA

**Keywords:** Endophytes, IAA, Orchid, *Vanda cristata*, Symbiosis

## Abstract

**Background:**

A plant growth-promoting endophytic bacterium PVL1 isolated from the leaf of Vanda cristata has the ability to colonize with roots of plants and protect the plant. PVL1 was isolated using laboratory synthetic media. 16S rRNA gene sequencing method has been employed for identification before and after root colonization ability.

**Results:**

Original isolated and remunerated strain from colonized roots were identified as Bacillus spp. as per EzBiocloud database. The presence of bacteria in the root section of the plantlet was confirmed through Epifluorescence microscopy of colonized roots. The in-vitro plantlet colonized by PVL1 as well as DLMB attained higher growth than the control. PVL1 capable of producing plant beneficial phytohormone under in vitro cultivation. HPLC and GC-MS analysis suggest that colonized plants contain Indole Acetic Acid (IAA). The methanol extract of Bacillus spp., contains 0.015 μg in 1 μl concentration of IAA. PVL1 has the ability to produce antimicrobial compounds such as ethyl iso-allocholate, which exhibits immune restoring property. One-way ANOVA shows that results were statistically significant at *P* ≤ 0.05 level.

**Conclusions:**

Hence, it has been concluded that Bacillus spp. PVL1 can promote plant growth through secretion of IAA during root colonization and ethyl iso-allocholate to protect plants from foreign infections. Thus, this study supports to support Koch’s postulates of bacteria establishment.

**Supplementary Information:**

The online version contains supplementary material available at 10.1186/s12896-021-00676-9.

## Introduction

Orchids are ornamental angiosperms and hidden nature’s treasure in all forms [1]. It has commercial value due to beautiful flowering part and possesses many scientific obscure [2, 3]. Habit of the plant allows specific microbiome in symbiotic association [4]. Orchids endophytes were co-evolved with other soil microbial community. The microbial communities inhabit orchid plants in symbiotic association that provides nutrient and enhance immunity to the orchid. The engineering of the colonization pattern of the microbes hold the entire ecosystem of the planet. Orchid seed lacks endosperms because of its genetic makeup [5]. In this connection, the fungal community fits the role of the endosperm providing carbon source for the seed germination. Therefore, fungal association is crucial for the seed germination and development. However, just few percentage of the seed dust gets chance to established mycorrhizal association for germination [4]. Moreover, there are endophytic bacteria possess important property for establishment of symbiotic association. Similarly, endophytic fungi such as *Rhizoctonia* sp., *Tullsenlla*, have been well explored for their orchid mycorrhizal nature. In nature, Orchids are in constant search of beneficial symbiotic pattern in existing natural places. They are few reports on orchid-bacteria interactions showing the potential roles of bacteria as a biological tool for the orchid conservation and bioactive compound production. However, some bacterial species along with fungi were considered as ideal source for studying orchid-microbes interaction and their impacts on development of orchid species [6].

The exploration of the beneficial or helper bacteria from the orchid species has the equal potential to conserve endangered orchid species [7–9]. However, few reports beneficial bacteria from the orchid species explained how bacteria interact with different orchid species. Potential applications of endophytic bacteria have been well studied in other plants. Especially, plant growth promoting rhizospheric bacteria (PGPR) were constantly reported as plant root colonizing endophytic bacteria that help plants in uptake of nutrient and minerals from soil [10, 11]. PGPR play an important role in mineralization of organic phosphorus, iron, fixation of di-nitrogen, production of plant growth hormone and plant growth regulator [12]. In this regard, the present study aims to isolate, characterize and reconfirms presence of leaf endophytic bacteria from the *Vanda cristata* (Orchid) that can colonise the roots of other orchid species; promote plant growth promotion and plant protection. *Vanda cristata* have become rare orchid species in forests of central Nepal [1]. It is an epiphytic orchid that grows in the braches of *Quercus* sp. and *Rhododendron* sp. The deforestation and recurrent fire in the forest have affected its natural habitat [1]. Moreover, these artificial calamities severely affected the regeneration and reestablishment of this plant. The present work describes how the bacterium residing in leaf of one orchid species can colonies the root across the genus and contribute for the fitness and growth of the plant? The study also works out to support the Koch’s postulates by re-isolating same bacteria from the root of the in-vitro bacterial colonized plantlet and again performing the plant assay with re-isolated of bacterium.

## Results

Bacterium isolated from roots of *Vanda cristata* (Orchid) was identified using 16S rRNA gene sequencing method belongs to phylum Firmicutes. Original isolated species and re-isolated species from colonized root was identified as *Bacillus* spp. Designation PVL1 and DLMB were given to original isolate and re-isolated species respectively. To avoid confusion bacterium has been termed as *Bacillus* spp., for original and re-isolated strain i.e. DLMB was a re-isolated form of PVL1 colonized plantlet. 16S rRNA gene sequencing method was adopted to identify *Bacillus* spp. Consensus sequence has been prepared using Lasergene SeqmanPro DNASTAR’s tool provided by National Centre for Microbial Resource, National Centre Cell Science (Pune). The original isolate employed for root colonization experiment and re-isolate from colonized root were same species with the 99 to 100% identity with type strain of *Bacillus subtilis* subsp*subtilis* NCBI 3610 T – Accession No. ABQL01000001 (Table 1). Identities of both bacteria were confirmed using 16S rRNA database called as EzBiocloud database (https://www.ezbiocloud.net/).

**Table 1:** Identification of endophytic bacteria isolated under this study

Morphotype	Tentative affiliation	Query coverage	Percentage of Identity	Accession code
PVL1	*Bacillu*s sp.	99%	99%	EU221672
DLMB	*Bacillu*s sp.	100%	100%	MW287212

### Quantification of IAA by HPLC technique

**Fig. 1:**
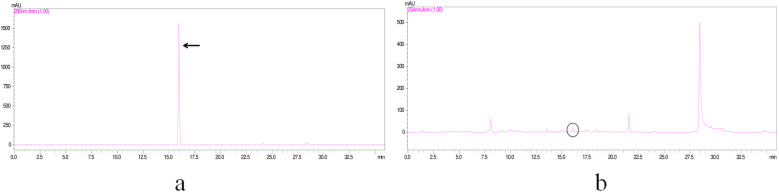
Quantification of IAA by Reverse-phase (RP) HPLC, the chromatograms (**a**) of the IAA standard 20 μL; 1 mg per mL (concentration) in comparison to those of endophytic bacteria PVL1; (**b**) The IAA (as indicated by arrows) was eluted with retention time of 10.2 min, indicated by arrow for standard and circle for PVL1 extract

Eighty milligram dry bacterial cell extract was suspended in 1 mL of HPLC grade methanol. Filtered methanolic extract was then used for quantification of IAA by HPLC technique. Sample injection volume in HPLC was 20 μL comprises 0.30 μg of IAA (Fig. 1).

### Detection of bioactive compounds

The metabolites present in bacterial extract were identified using GC-MS. The presence of plant phytohormone IAA shows its role in root development and plant growth and bioactive metabolites such as Hexadecanoic acid, Ethyl iso-allocholate, Cyclopropane, 12-Methyl-oxa-cyclododecan-one, 6-Acetyl-beta-d-mannose were detected in bacterial extract (Table 2). Amongst bioactive metabolite Hexadecanoic acid, Ethyl iso-allocholate, Cyclopropane possesses antimicrobial property and reported to overcome biotic stress on plant and contribute to the fitness of the plant (Table 1 and Supplementary File 1).

**Table 2:** GC-MS mediated detection of bioactive compounds using present in bacterial methanolic extract

Peak	Retention Time	Name of compound	Base Peak	Reported Biological function
1	3.514	6-Acetyl-beta-d-mannose	43.10	Involved in plant metabolism, Cell wall component [13]
2	8.901	1H-Indole-3-acetic acid	130.25	Plant and root growth and Development [14]
3	9.549	n-Hexadecanoic acid	41.05	Biotic stress tolerant [15]
4	10.746	Oleic Acid	41.05	Embryo development [16]
5	14.011	Ethyl iso-allocholate	43.10	Biotic stress tolerant [17]
6	16.270	Cyclopropane	43.05	Drought tolerant [18]

### Detection of bioactive compounds present in non-colonized and colonized plant extract

GC-MS analysis of extract obtained from uncolonized and colonized plant roots contain Ethyl iso-allocholate, d-Mannitol, 1-O-(22-hydroxydocosyl)-, saturated fatty acid viz. Hexadecanoic acid, Heptadecanoic acid, Octadecanoic acid in untreated plant extract (Table 3). The bioactive compounds present in DLMB bacteria colonized plant were 1H-Indole-3-acetic acid, methyl ester, Eicosanoic acid (Table 3). The compound present in PLV1 colonized plant extract were 1H-Indole-3-acetic acid, methyl ester, Palmitic acid, Octadecanoic acid, Digitoxin, d-Mannitol, L-Ascorbic acid and Ethyl iso-allocholate (Table 4). Among these three extract Hexadecanoic acid Octadecanoic acid, d-Mannitol were common bioactive compounds whereas Indole-3-Acetic Acid (IAA) and Methyl Ester was present in both treated plantlet. Moreover, Ethyl iso-allocholate compound was present in both DLMB plant extract and untreated plant extract (Tables 2 and 3). Eicosanoic acid only present in DLMB bacteria colonized extract (Table 4); L-Ascorbic acid, Digitoxin extract were only present in PVL1 bacteria colonized extract (Table 5). Showing the various bioactive compounds in their different metabolite studies (Supplementary File 1).

**Table 3:** List of the various compounds identified by GC-MS analysis present in the untreated plant extract

Peak	Retention Time	Name	Base Peak	Reported biological function
1	10.410	Hexadecanoic acid, methyl ester	74.10	Antimicrobial activity [15]
2	11.125	Heptadecanoic acid, heptadecyl ester	43.15	Antimicrobial activity [15]
3	12.930	Hexadecanoic acid, 1-(hydroxymethyl)-1,2-ethanediyl ester	57.15	Antimicrobial activity [15]
4	13.580	d-Mannitol, 1-O-(22-hydroxydocosyl)-	73.10	Role in abiotic and biotic stress [14]
6	17.850	Ethyl iso-allocholate	55.10	Antimicrobial acitivity [17]
7	19.445	d-Mannitol, 1-O-(22-hydroxydocosyl)-	73.10	Role in abiotic and biotic stress [19]
8	28.080	Ethyl iso-allocholate	43.15	Antimicrobial acitivity [17]

**Table 4:** List of the various compounds identified by GC-MS analysis present in the (DLMB) colonized plant extract

Peak	Retention Time	Name	Bass peak	Reported biological activities
1	9.990	1H-Indole-3-acetic acid, methyl ester	130.15	Important plant hormone and signaling molecule for overall plant growth and development [14]
2	10.705	Eicosanoic acid	73.10	Plant defense against microbes [20]
3	10.400	Hexadecanoic acid, methyl ester (Palmatic acid)	74.10	Antimicrobial Activities [15]
4	10.535	Octadecanoic acid	55.10	Plant self defense mechanism against plant pathogenic fungi [21, 22]
5	11.865	d-Mannitol, 1-O-(22-hydroxydocosyl)-	55.15	Play an important role against biotic and abiotic stress [14]
6	11.565	Oleic acid	55.10	Seed development [16]; biotic stress tolerant [21, 22]

**Table 5:** List of the various compounds identified by GC-MS analysis present in the PVL1 colonized plant extract

Peak	Retention Time	Name	Bass peak	Reported biological activities
1	10.015	1H-Indole-3-acetic acid, methyl ester	130.15	Important plant hormone and signaling molecule for overall plant growth and development [14]
2	10.405	Palmitic acid, methyl ester	74.10	Biotic stress tolerant [15]
3	10.065	1H-Indole-3-acetic acid	130.15	Important plant hormone and signaling molecule for overall plant growth and development [14]
4	11.705	Octadecanoic acid	74.10	Plant self defense mechanism against plant pathogenic fungi [21, 22]
5	11.565	Ethyl iso-allocholate	44.10	Antimicrobial activity of plant against pathogen [17]
6	14.610	Digitoxin	44.10	Induces cytotoxicity of cancer cell line [23]
7	13.490	d-Mannitol, 1-O-(22-hydroxydocosyl)-	44.05	Important role in abiotic and biotic stress [19]
8	28.680	L-Ascorbic acid 6-palmitate	44.10	Important factor in biological pathway and plant photosynthesis [24, 25].

### Plant growth assay

**Fig. 2:**
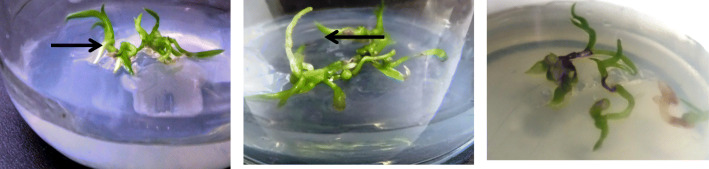
In-vitro plant growth assay of *Cymbidium aloifolium,* **a** plantlet interacted with PVL1 **b** Plantlet interacted with DLMB; **c** control

**Fig. 3:**
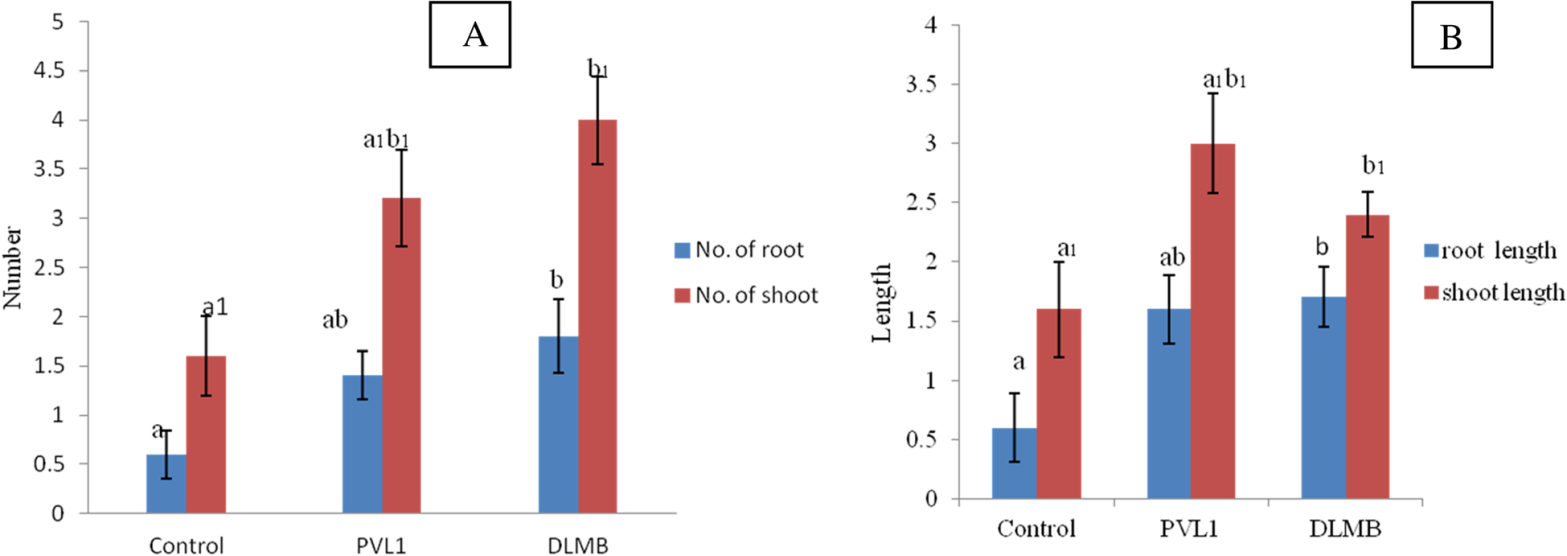
The morhopogical status of *Cymbidium aloifolium* plantlets treated with different endophytic bacteria (*n* = 10). **a** Number of roots and shoots; **b** Mean root and shoot length in cm; **c** Values with different letters are significantly different at *p* ≤ 0.05 (Tukey test)

The *Cymbidium aloifoium* in-vitro plantlets on Tryptophan supplemented MS media were interacted with bacteria DLMB and PVL1 isolated from *Vanda Cristata*. The plant growth was observed for 45 days with respect to control (untreated) plantlet. The plantlet interacted with the DLMB and PVL1 showed significantly higher growth than the control as shown in the graph figure. Among the control, DLMB and PVL1, the length of shoot and root was higher in the plantlets treated with DLMB whereas the number of root and shoot were higher in the plantlets treated with PVL1. The figure shows the interaction of DLMB and the PVL1 with *Cymbidium aloifolium*. The plantlet treated with DLMB PVL1 shows multiple roots and shoots formation as compare to control (Figs. 2 & 3).

### Confirmation of root colonization

**Fig. 4:**
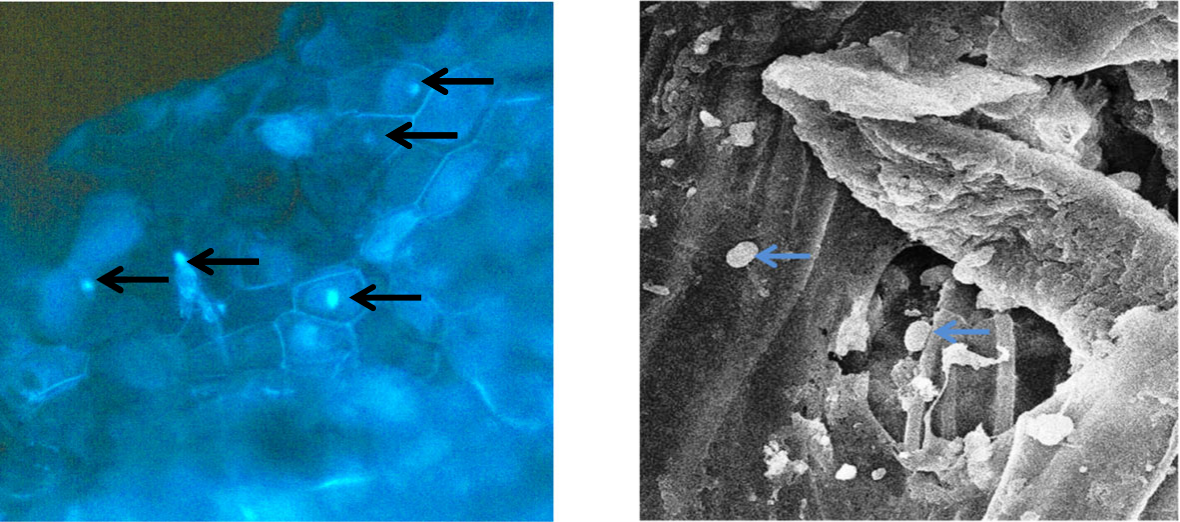
Epiflorescene microscopy. Free hand cut root section colonized by bacteria PVL1 treated with 90% alcohol for 5 min followed by DAPI staining. The arrow indicates bacteria colonized inside plant cell. Scanning electron microscopy showing the presence of rod shaped bacteria in the bacteria colonized root section

Histochemical analysis of the root section of the plantlet interacted with PVL1 and DLMBX was performed by DAPi staining as shown in Fig. 4. The detection of rod shaped bacterium in the root section interacted was further confirmed the symbiotic association by Scanning Electron microscopy Fig. 4.

## Discussion

In this investigation, endophytic bacterium has been isolated from Nepal occupied Himalayas Orichid (*Vanda cristata*) identified as *Bacillus spp.,* strains PVL1 and DLMB. PVL1 was the original isolate whereas DLMB was artificially infected PVL1 strain called as re-isolated strain and designated as DLMB. Both strains were shows 99–100% identity with 16S rRNA gene sequence of type strain *Bacillus subtilis* subsp. *subtilis* NCIB 3610 T (ABQL01000001). Both isolates were potential candidates and promote growth of *C. aloifolium*. The result from plant growth assay as well as metabolimic study of both plant extract (uncolonized and colonized) and bacterial methanol extract reveals the significant contribution of bacteria toward plant growth. Previous study shows the endophytic bacteria, *Bacillus substillis* have also been reported to plant growth promoter and regulator [26–29]. *B. substillis* shows symbiotic interaction with *Lonicera japonica*. Interactions with *L. japonica* have increase in plant growth such as shoot, root and chlorophyll content. The study also revealed the ability of *B. substillis* to produce IAA, cellulose, pectinase enzyme as well as inhibit pathogen such as *F. oxysporum*. Similarly study, clearly explained the significant role of *B. substillis* AlB629 when interacted with cacao seedling. Moreover, increased growth has provided immunity against pathogen attack [30]. The *B. substillis* HC8 isolated from giant hogweed also reveal its symbiotic nature when interacted with tomato seedling [31]. The seedling infected with *B. substillis* HC8 not only promoted high growth of the seedling but also showed resistant against tomato foot and root rot. In metabolic study, the presence of growth promoting volatile compounds and hormone such as gibberelin and (lipo) peptide as antibiotic were revealed [32, 33]. Similar experiment, showing both plant growth promoting as well as pathogen resistance provided by *B. amylblignefacien* when interacted with tomato seedling. The treated plantlet showed increased amino acid metabolism, higher salicylic acid production [28].

*Bacillus* spp., isolated from orchid proved to be plant tissue colonizing nature bacterium. *D. nobile* seed when interacted with *B. pumilus* colonized the seed of *D. nobile* [34]. similar experiment with *B. substilis* and epiphytic orchid *Anoectochilus Roxburghii* and *A. Formosanus* has proven that *B. substilis* not only promoted the growth of in- virto plantlet but also enhance the production of flavonoids, steroids and essential oil [35]. These studies have shown the plant growth promoting potentiality of *Bacillus* spp. In the current investigation, 45 days of the plant growth assay performed by interacting *C. aloifolium* and PVL1 as well as DLMB (re-isolated form of PVL1 colonized plantlet) have proven the beneficial effect of PVL1 (*B. substilis*). The plant colonized by both PVL1 and DLMB (re-isolated from PVL1 colonized plantlet) attend two-fold growth in terms of shoot and root length as well as their number as compare to control.

Overall facts reveal the plant when interacted with PVL1 and DLMB attend growth and development showing resistant to abiotic and biotic stress. This can be further explained by the biochemical assay experiments. The production of IAA by PVL1 as detected and quantified by using HPLC technique. The production of IAA in concentration of 0.015 μg per μL, when supplemented 2 mg of DL-Tryptophan in 20 mL of broth shows its capacity to produce IAA. The bacterial ethyl acetate extract contain numerous phytohormone and plant growth factor that have play major role in plant growth has been proven by characterization of metabolite present in both plant colonized and bacterial extract. The bioactive compounds identified from the bacterial extract, non-colonized plant extract and PVL1 and DLMB colonized extract clearly explains the bacterial produce some important plant hormone, growth factor and growth regulators that promotes the growth of the plant. Similarly, the detection of antioxidant as well as molecules contributes to the fitness of the plant against pathogen and abiotic stress. Apart from IAA, the presence of plant metabolites such as 6-Acetyl-beta-d-mannose, Cyclopropane L-Ascorbic acid 6-palmitate, Eicosanoic acid, d-Mannitol, 1-O-(22-hydroxydocosyl)-, Ethyl iso-allocholate and Oleic acid are some important bioactive compounds that help to overcome both abiotic and biotic factors [13–25]. Among these Oleic acid is detected in bacterial extract as well as in bacteria colonized plant extract. Whereas Mannitol, L-Ascorbic acid was only seen in both PVL1 and DLMB colonized plant extract. This indicates the bacterial has helped the in-vitro plantlet to trigger its own immune system. Whereas Cyclopropane is one of the important precursor biomolecules for the production of plant hormone ethylene was detected bacterial extract. The DAPI staining of the root treated with bacteria show the presence of bacteria in the cell of the root section. The rod shaped bacteria were detected in scanning electron microscopy that further confirms the root treated with bacteria was successfully colonized. More importantly, the bacterium isolated from the leaf section of Vanda cristata were able to colonise the roots of *Cymbidium aliofolium*, proving to be root colonizing bacteria across the genus. This evidences supports the symbiotic nature of the bacterium PVL1 and can have potential biotechnological application.

## Material and methods

A leaf of *Vanda cristata* was collected in sterile sampling bag without damaging the plant population. The root section was then brought under cooling condition in icebox to the Plant Biotechnology Research Laboratory, Central Department of Botany, Tribhuvan University.

### Isolation of root colonizing endophytic bacteria

The leaf section was surface sterilized according the protocol used by Shah et al. (2019) [36]. The surface adhere microbes were washed out in running tap water in presence of Tween 20 for 20 min. The leaf section was treated with 75% ethanol 1 min and then by 3% Sodium hypochloride (NaOCl) for 10 min, followed by 90% ethanol treatment for 30 s. Finally, rinsed three times by autoclaved distilled water. The leaf section was cut in small sections of about 1 cm by sterile scalpel. After complete surface sterilization the root section were placed in synthetic media. Leaf section from *Vanda cristata* were placed in Potato Dextrose media and incubated at 28 °C for 7 days. Screening for endophytic bacteria have been carried out and pure culture of was saved on sterile media plate designated as PVL1. Additionally, glycerol stocks were prepared for long-term storage under low temperature.

### DNA extraction, PCR amplification and sequencing of 16S rRNA gene

The genomic DNA extraction has been extracted by CTAB method. TaKaRa bacterial 16S rDNA PCR Kit Fast (800) was used for 16S rRNA gene sequecning (TaKaRa Bio Inc.; Cat. # RR182A). The genomic DNA extraction has been extracted by CTAB method. 16S rRNA gene sequencing of PVL1 has been carried out using two pairs of forward and reverse primers [27F, 5′- AGAGTTTGATCCTGGCTCAG - 3′; 1392R: 5′- GGTTACCTTGTTACGACTT - 3′; 536F: 5′- GTGCCAGCMGCCGCGGTRATA - 3′ and 907R: 5′- CCGTCAATTCMTTTGAGTTT - 3′]. PCR condition used for amplification of the 16S rRNA gene were: Initial denaturation 4 min at 95 °C, denaturation at 95 °C for 60 s, annealing at 55 °C for 60 s, extension at 72 °C for 60 s and final extension at 72 °C for 7 min. Total 35 cycles has been set to obtain good yield of amplified DNA along with positive control and negative control. Consensus sequences of amplified DNA from individual primers has been prepared using Lasergene SeqmanPro DNAStar’s standalone tool. The consensus sequence was submitted to the Genebank database to obtain accession number. Bacterial sequence has been identified using EzBiocloud- an ideal website for identification of bacteria. Phylogenetic analysis was carried out using MEGA X standalone software on Mac OS X 10.13.6.

### Plant growth assay

The in-vitro grown plantlet (Protocorm turn small plantlet stage) was taken for the plant growth assay experiment. The plantlets were allowed to grow in full MS media supplemented with 2 mg/L of DL Tryptophan (Hi-media, India) of media. The bacterial colony was inoculated on surface of the media just next to the plantlet. The control contains five replicates were taken and same parameter of light and nutrient was provided. The plant growth assay was kept for the observation of 2 months. The root and shoot number as well as length were calculated to find the significant differences between the colonized and non-colonized plant. Aseptic conditions were maintained during the plant growth assay for 2 months under a 16 h photoperiod, and at 25 ± 2 °C.

### Confirmation of endophytic bacterium

The bacteria inoculated during the plant growth assay were re-isolated and identified using 16S rRNA gene sequencing method. Strain was re-isolated from cultured root contain colonized of bacteria which can be observed through scanning electron microscopy. This re-isolated strain designated as DLMB obtained from plant growth assay after surface sterilization of root section. Thus, experiment was performed to show plant growth assay and re-isolation of same bacteria. After completion of growth assay from root cross-section has been reanalyzed to prove bacterium PVL1 (new re-isolated strain DLMB) possess symbiotic nature of bacteria supporting the Koch’s postulates.

### Biochemical assay

Biochemical assay was performed to estimate the concentration Indole compound (plant growth) present in bacterial extract.

### Quantification of IAA

Bacterium PVL1 was inoculated in 20 ml Czapek Dox broth supplement with and without DL Tryptophan (1 mg). The broth was incubated at 28 °C for 10 days for the growth of bacteria. The broth was centrifuged at 9660 g for 10 min at 18 °C. The supernatant was treated with few drop of 1 N HCl to obtain cell-free extract. Treated cell free extract was filtered using Whatman filter paper Grade 1: 11 μm (medium flow filter paper). For the quantification of the IAA, one millilitre (1 mL) filtrate was treated with 2 mL of Salknowski reagent (Brick, Bostock and Silverstone 2004). Thereafter, it was incubated at dark for 20 min. The optical density was measured at 530 nm using an UV-VIS spectrophotometer (ChromTech, Model CT 8200). The concentration per mL was calculated with help of standard IAA curve. The experiment was replicated three times to verify the concentration of IAA. Remaining 19 mL extract was supplemented with DL Tryptophan and used for detection of IAA bioactive compound by High Performance Liquid Chromatography (HPLC) and Gas Chromatography-Mass Spectrometry. For HPLC and GC-MS analysis, filtrate was washed three times with equal volume of ethyl acetate. The mixture of ethyl acetate and bacterial broth was allowed withstand for an hour ina separatory funnel. The organic layer was saved. Solvent has been evaporated using a rotary evaporator (DLAB: RE100-Pro) at 40 °C. The dry weight of the extract was 80 mg. The extract was dissolved in 1 mL methanol (HPLC grade). The extract was filter by using 0.22-μm filter. The filtrate was used for quantification of IAA by HPLC technique and bioactive compound characterization by GC-MS technique.

### Confirmation for IAA by HPLC

Samples were analyzed with HPLC (Shimaduza UFLC, Model DGU-20 A5R, Japan). Analysis conditions were: C18 shim- pack column size (5μmX 4.6X150mm); oven temperature at 30 °C throughout the experiment; absorbance of IAA at 280 nm; mobile phase 0 to 55% acetonitrile for 25 min. Then 100% acetonitrile for 28 min. 0.1% acetic acid in water flow rate 1.5 mL per min. The 20 μl of sample was injected. Retention times for compound were compared to those of standard graph of IAA prepared.

### Detection of bioactive compound

The presence of the bioactive compound in the bacterial organic extract was done by using GC-MS technique as per previously reported [35]. The instrument used for the experiment was GC-MS (QP2010 Ultra, Shimadzu Europa GmbH, Germany) and the column used was RTX-5MS (30 × 0.25 × 0.10 m). The increase in temperature was 3 °C min^− 1^ whereas the initial and final temperature of the instrument was set to 100 °C and 250 °C respectively. The peak obtain in chromatograph was identified as tentative bioactive compound by comparing with library of National Institute of Standard and Technology, NIST, US.

### Comparative chemical profiling of colonized and non-colonized plantlet

Three months old in-vitro plantlet was taken to prepare the methanol extract. Colonized and non-colonized plantlets were ground in separately in an autoclaved mortal and pestle. The 20 mL HPLC graded methanol was added to the extract to dissolve all the compounds present in the plantlet extract. Further the extract was filtered using filter paper and the volume of methanol was reduced to 3 mL to concentrate the extract and metabolites. The extract was then used for the identification of the compounds by GC-MS technique as described above as described in previous report [37].

### Epifluorescence microscopy of colonized root section

The root sections of the colonized plant are taken to investigate the presence of the bacteria spore inside the root tissue. In this regard, the specimen preparation and staining procedure was followed as described by Thomas and Reddy (2013). Thin section of the root tissue was treated with for 90% ethanol followed by (4, 6-Diamidino-2-phenylindole) DAPI staining. Scanning electron microscopy of dehydrated to root section has been according to method described by Thomas and Reddy (2013) [38].

### Statistical data analysis

The plant growth assay was calculated in term of shoot and root length as well as their number. For the plant growth assay, was performed independently with five replicates to measure the shoot and root length and number. The significance of data was tested by one-way ANOVA with the alpha error level set at *P* ≤ 0.05 (Tukey HSD test).

## Supplementary Information

**Additional file 1.**

## Data Availability

All data generated and analysed are available in this published article and the Supplementary data file 1.
