# Supplementary material for: A prospectus of plant growth promoting endophytic bacterium from orchid (Vanda cristata)
Source: BMC Biotechnol. 2021 Feb 22;21:16. doi: 10.1186/s12896-021-00676-9 (PMC7901085; doi:10.1186/s12896-021-00676-9)
Supplement: Supplementary file 1 — Additional file 1. [file 12896_2021_676_MOESM1_ESM.docx]

**A prospectus of plant growth promoting endophytic bacterium from Orchid (*Vanda cristata*)**

Sujit Shah^1,4*^, Krishna Chand^1^, Bhagwan Rekadwad^2^, Yogesh S. Shouche^2^, Jyotsna Sharma^3^, Bijaya Pant^1^*

^1^Central Department of Botany, Tribhuvan University, Nepal

^2^National Centre for Microbial Resource, National Centre for Cell Science, Savitribai Phule Pune University Campus, Ganeshkhind, Pune 411021, India

^3^Department of Plant Science, Texas Tech University, USA

^4^ Daffodil Agro Biological Research Center, Nepal

*Corresponding authors email addresses: [b.pant@cdbtu.edu.np](mailto:b.pant@cdbtu.edu.np) [sujitaug16shah@gmail.com](mailto:sujitaug16shah@gmail.com)

**Supplementary Figure 1: The GCMS chromatogram of the methanol extract of uncolonised plant**

**Supplementary Table1: The list of the compounds identified from methanol extract of uncolonised plant**

| Peak | Retention Time | Name | Mass peak | Base Peak |
| --- | --- | --- | --- | --- |
| 1. | 10.410 | Hexadecanoic acid, methyl ester | 640 | 74.10 |
| 2. | 11.125 | Heptadecanoic acid, heptadecyl ester | 586 | 43.15 |
| 3. | 12.930 | Hexadecanoic acid, 1-(hydroxymethyl)-1,2-ethanediyl ester | 588 | 57.15 |
| 4. | 13.580 | d-Mannitol, 1-O-(22-hydroxydocosyl)- | 613 | 73.10 |
| 6. | 17.850 | Ethyl iso-allocholate | 685 | 55.10 |
|  |  |  |  |  |
| 7. | 19.445 | d-Mannitol, 1-O-(22-hydroxydocosyl)- | 704 | 73.10 |
| 8. | 25.975 | beta. Carotene | 820 | 55.10 |
| 9. | 28.080 | Ethyl iso-allocholate | 685 | 43.15 |

**Supplementary Figure 2: The GCMS chromatogram of the methanol extract of colonised plant by PVL1**

**Supplementary Table2: List of the compounds identified from methanol extract of plant colonized by PVL1**

| Peak | Retention Time | Name | Mass peak | Bass peak |
| --- | --- | --- | --- | --- |
| 1. | 10.015 | 1H-Indole-3-acetic acid, methyl ester | 562 | 130.15 |
| 2. | 10.405 | Palmitic acid, methyl ester | 574 | 74.10 |
| 3. | 10.065 | 1H-Indole-3-acetic acid | 558 | 130.15 |
| 4. | 11.705 | Octadecanoic acid | 576 | 74.10 |
| 5. | 11.565 | Ethyl iso-allocholate | 576 | 44.10 |
| 6. | 14.610 | Digitoxin | 596 | 44.10 |
| 7. | 13.490 | d-Mannitol, 1-O-(22-hydroxydocosyl)- | 595 | 44.05 |
| 8. | 28.680 | L-Ascorbic acid 6-palmitate | 548 | 44.10 |

**Supplementary Figure 3: The GCMS chromatogram of the methanol extract of colonized by DLMB**

**Supplementary Table3: List of the compounds identified from methanol extract of colonised plant by DLMB**

| Peak | Retention Time | Name | Mass peak | Bass peak |
| --- | --- | --- | --- | --- |
| 1. | 9.990 | 1H-Indole-3-acetic acid, methyl ester | 445 | 130.15 |
| 2. | 10.300 | Cinnamic acid, | 442 | 55.15 |
| 3. | 10.705 | Eicosanoic acid | 451 | 73.10 |
| 4. | 10.400 | Hexadecanoic acid, methyl ester (Palmatic acid) | 460 | 74.10 |
| 5. | 10.535 | Octadecanoic acid | 442 | 55.10 |
| 6. | 11.865 | d-Mannitol, 1-O-(22-hydroxydocosyl)- | 457 | 55.15 |
| 7. | 11.565 | Oleic acid | 457 | 55.10 |

**Supplementary Figure 4: The GCMS chromatogram of the methanol extract of PVL1 (Bacteria)**

**Supplementary Table 4: List of the compounds identified from methanol extract of PVL1 (Bacteria)**
